# From Biomarker to Trigger: Biological Calibration of Stimuli-Responsive Gels Toward Precision Periodontal Therapy

**DOI:** 10.3390/gels12100897

**Published:** 2026-10-03

**Authors:** Lovre Martinović, Roko Šantić, Ena Kuliš, Dinko Martinović, Laura Jurina Kumrić, Marko Kumrić, Joško Božić

**Affiliations:** 1 Department of Pathophysiology, University of Split School of Medicine, 21000 Split, Croatia; martinlovre@gmail.com (L.M.); roko.santic@mefst.hr (R.Š.); marko.kumric@mefst.hr (M.K.); 2 Laboratory for Cardiometabolic Research, University of Split School of Medicine, 21000 Split, Croatia; 3 Department of Dental Medicine, University Hospital of Split, 21000 Split, Croatia; 4 Department of Maxillofacial Surgery, University Hospital of Split, 21000 Split, Croatia; 5 Department of Maxillofacial Surgery, University of Split School of Medicine, 21000 Split, Croatia; 6 Implant Centar Markic, 21000 Split, Croatia

**Keywords:** functional gels, stimuli-responsive hydrogels, periodontitis, gingival crevicular fluid, biological calibration, local drug delivery, matrix metalloproteinase-8, periodontal microenvironment

## Abstract

Endogenous stimuli-responsive gels are designed to alter drug release or matrix behavior in periodontal sites, but their responsiveness is often demonstrated under conditions that may not represent the biological compartment encountered after placement. This comprehensive review examined whether proposed periodontal triggers have been biologically calibrated. Targeted searches of PubMed/MEDLINE, publisher websites, and backward and forward citations identified human studies measuring periodontal pH, proteases, redox conditions, oxygen tension, microbial metabolites, and glucose, together with gel studies using the same cues. Human pH measurements ranged from mildly acidic to alkaline and did not support a universal activation window. Protease-responsive systems had the strongest mechanistic rationale, although recombinant-enzyme testing was not equivalent to native-fluid cleavage. Hydrogen peroxide-responsive gels were generally evaluated under challenge conditions above the limited direct gingival crevicular fluid evidence and without physiological antioxidant buffering. Oxygen-generating systems did not directly sense local oxygen tension. Some glucose challenges overlapped reported human GCF measurements, but calibration to defined periodontal sites and treatment conditions remained unverified. Across trigger classes, preclinical efficacy seldom isolated responsiveness from payload and carrier effects. Future studies should use compartment- and treatment-matched measurements, realistic input–output testing under flow and buffering, patient-derived fluids, and controls that isolate cue-dependent material behavior.

## 1. Introduction

The current classification of periodontitis separates accumulated severity and case complexity (stage) from estimated progression and risk modifiers (grade). Although this framework can accommodate future biomarker refinement, grading does not define a molecular state that can directly control a drug-delivery material [1]. At an individual site, probing depth, bleeding, attachment loss, bone loss, smoking, and glycemic control remain clinically informative but do not specify the concentration, activity, or persistence of a cue at the gel surface. Autonomous responsiveness therefore requires evidence beyond diagnostic or prognostic association.

Within the European Federation of Periodontology stepwise pathway, the first step comprises patient engagement, supragingival biofilm control, professional mechanical plaque removal, and risk-factor management; subgingival instrumentation is the core second step for stage I–III periodontitis [2]. Local or systemic adjuncts are then selected according to clinical presentation and treatment response. Functional gels are attractive adjunctive depots because they conform to confined sites and retain payloads that would otherwise be cleared rapidly. Endogenous stimuli-responsive systems make a stronger claim: a disease-associated cue should preferentially alter release, erosion, or another material function. Existing reviews catalogue systems responsive to pH, reactive oxygen species (ROS), proteases, glucose, or multiple stimuli according to chemistry, therapeutic function, or healing phase [3,4,5], but do not establish that the applied experimental inputs reproduce those encountered in human periodontal pockets.

Periodontal delivery materials extend beyond hydrogels to lipid vesicles, polymeric particles, inorganic nanomaterials, and dendritic carriers. Liposomes provide lipid-bilayer and aqueous compartments for payload incorporation, whereas poly(lactic-co-glycolic acid)/chitosan nanospheres have been investigated for local delivery of metronidazole or the host-modulating agent N-phenacylthiazolium bromide [6,7]. Inorganic components can supply an additional therapeutic function: zinc oxide nanoparticles and minocycline have been incorporated into albumin particles dispersed in a Carbopol hydrogel [8]. Highly branched dendrimers offer multiple functional groups for cargo association; spermine-modified glucan dendrimer gels delivered small interfering RNA targeting osteoclast stimulatory transmembrane protein (OC-STAMP) and reduced bone resorption in a mouse periodontitis model [9]. These examples illustrate different loading and delivery architectures, with predominantly preclinical evidence. Incorporating a nanocarrier into a macroscopic gel adds a retention matrix and can introduce sequential release barriers, governed by particle-payload interactions and transport through or erosion of the surrounding network. The biological input may act on either component, so the location of the responsive element must be specified.

The rationale for a local depot must be distinguished from evidence that endogenous responsiveness improves treatment. Meta-analyses of locally delivered antimicrobials and gel adjuncts reported small or short-term mean improvements with substantial heterogeneity among formulations and studies [10,11]. These findings support adjunctive delivery, not the superiority of an endogenous switch. Targeted local delivery may reduce systemic exposure and administration frequency, but excessive or inappropriate local antibiotic use may still select for resistance within oral biofilms [12]. A responsive system must demonstrate adequate local exposure and show that the proposed cue materially changes that exposure.

The term responsive denotes an experimentally observed input–output relation. A gel may release more payload at pH 5.5 than at pH 7.4, degrade in peroxide, or be cleaved by recombinant matrix metalloproteinase-8 (MMP-8), yet these tests do not necessarily reproduce exposure after clinical placement. Biological calibration requires a cue in a molecular form accessible to the response chemistry, at a magnitude, duration, and location that intersect the material response and produce a therapeutically relevant output. The comparison must involve the compartment in direct contact with the gel. Oral-rinse thresholds, salivary damage markers, or antigen measured in diluted gingival crevicular fluid (GCF) cannot be transferred directly to a pocket-level cleavage threshold. Likewise, animal efficacy does not demonstrate endogenous triggering without local cue measurements and controls that distinguish cue-dependent material behavior from carrier and payload effects.

This narrative review was informed by the Scale for the Assessment of Narrative Review Articles [13]. The original PubMed/MEDLINE search, completed through 22 August 2026, combined periodontitis, periodontal pocket, GCF, or subgingival with hydrogel, gel, nanogel, stimuli-responsive, stimulus-responsive, or microenvironment-responsive. Cue-specific searches covered pH, ROS/H_2_O_2_, MMP-8/MMP-9, other matrix metalloproteinases, elastase, gingipains, glucose, hypoxia/oxygen tension, hydrogen sulfide/volatile sulfur compounds, SCFAs, cytokines, and lipopolysaccharide. Citation chaining and targeted Google Scholar and publisher searches supplemented this core search. During revision, targeted searches through 20 September 2026 added evidence on carrier chemistry, plaque composition, and biodegradation, together with official regulatory and standards sources. PubMed search strategies are provided in Table S1.

The calibration synthesis included human measurements in defined periodontal compartments and periodontal gels whose endogenous inputs altered degradation, swelling, mechanics, transport, release, or chemical conversion. Eligible systems contained a functional hydrated polymer network as a bulk gel, crosslinked nanogel, or hydrogel shell, with responsiveness in the network or an incorporated carrier. Nanoparticles without a gel phase, purely thermogelling systems, and externally actuated systems were excluded from the calibration synthesis; selected carriers provided design context only. Thermogelation was considered a delivery mechanism rather than disease-selective activation; thermogels with an additional endogenous response remained eligible. Sialidase was discussed within human biomarker panels but was not assessed as a separate material trigger. Peri-implantitis was excluded to retain a tooth-associated periodontal scope without assuming equivalent exposure at implant interfaces. Records were assessed iteratively by title and abstract and by full text when relevant or uncertain. Extraction covered clinical state, compartment, sampling and assay, analyte form and magnitude or activity, treatment timing, material chemistry, challenge conditions, response kinetics, flow and native-fluid testing, and specificity controls.

Comparisons remained at study level, without pooling different compartments or equating enzyme antigen with catalytic activity, or oxidative-damage markers with free oxidant. Direct human measurements and clearly defined assays and compartments were prioritized. Complete primary full texts were not assessed for three material studies [14,15,16]. Their interpretation was restricted to accessible abstracts, publisher text, and verifiable information or original figures reproduced in subsequent publications. Where essential challenge or material-response data could not be verified, biological calibration was designated Not assessable. The review did not use duplicate screening, exhaustive systematic screening, formal risk-of-bias or GRADE assessment, meta-analysis, or a PRISMA flow diagram; screening counts were not recorded. The biological calibration categories were assigned jointly by two authors using the operational definitions: unresolved, unverified, limited, partial, and not assessable. Assessment considered the compartment in direct contact with the gel, the molecular form or functional activity measured, and the correspondence between human exposure and the experimental conditions shown to alter material behavior. Differences in interpretation were resolved through discussion and re-examination of the relevant source reports until consensus was reached. These categories were developed for this review and have not been externally validated; they describe biological calibration rather than study quality or therapeutic efficacy.

## 2. Material Chemistry, Properties, and Biodegradation

### 2.1. Network Chemistry and Material Properties

A responsive periodontal gel combines a hydrated polymer network with chemical elements that couple an input to altered network behavior, payload availability, or therapeutic function. Natural-polymer derivatives provide chemically accessible amine, carboxyl, or hydroxyl groups: carboxymethyl chitosan can react with oxidized dextran through imine linkages [17], whereas phenylboronic-acid-modified hyaluronic acid forms dynamic boronate ester crosslinks with poly(vinyl alcohol) (PVA) [18]. Synthetic poly(ethylene glycol) (PEG) networks allow protease-cleavable peptide segments to be introduced as defined crosslinks [19]. Thus, the polymer backbone and the responsive junction are separate design choices. Their coupling determines whether stimulation predominantly changes swelling, network integrity, or drug attachment, and whether an intact carrier, soluble polymer fragment, or free drug leaves the depot.

Polymer concentration, chain length, functionalization, and crosslink density determine network mechanics and transport. Controlled PEG experiments showed that crosslinking density and protein size alter diffusion, demonstrating that release cannot be inferred from trigger chemistry alone [20]. For periodontal deployment, gelation time, injection force, recovery after shear, wet adhesion, and swelling must be considered alongside release. For example, a hyaluronic-acid/PVA boronate gel required a maximum injection force of 21.88 N at 2 mL/min through a 0.5 mm × 20 mm needle and showed mean adhesion strengths of 9.07 kPa to porcine gingiva and 8.40 kPa to enamel [18]. These are formulation- and test-specific measurements rather than clinical acceptance thresholds. Increasing network cohesion may improve depot persistence while restricting payload transport or enzyme access; calibration should therefore characterize the hydrated network throughout its response, including residual off-state release.

### 2.2. Biodegradation During Treatment

Biodegradability should be evaluated separately from disappearance of the visible gel. Backbone scission, cleavage of crosslinks, dissolution, and physical washout can all reduce depot size, but produce different residual materials. Enzymatic degradation of chitosan-derived networks is possible, yet chemical modification can leave persistent crosslink-containing fragments: lysozyme-treated chitosan-genipin hydrogels retained some crosslink structures after network breakdown in an experimental degradation study [21]. Similarly, cleavage of peptide junctions in a PEG network or oxidation of boronate junctions in a PVA network does not by itself demonstrate complete degradation of the polymer backbone [19,22]. Solubilization should therefore not be equated with metabolism or safe elimination. The identity, molecular size, local persistence, and toxicity of released polymers, linker fragments, and inorganic components require formulation-specific evaluation. Safety testing should assess redox and pH changes during activation and the effects of degradation products on epithelial integrity and healing in inflamed, vascularized periodontal tissues.

Available periodontal studies demonstrate strongly formulation-dependent degradation. The embelin carrier prepared from carboxymethyl chitosan and oxidized dextran at a 10:1 volume ratio gelled in 55 ± 3 s and showed approximately 60% degradation in PBS over 25 days; alternative mixing ratios degraded more extensively over the same interval [17]. The PVA/boronate system discussed below instead degraded on markedly different timescales at 100 µM and 1 mM H_2_O_2_ [22]. Neither static-buffer degradation nor disappearance under an oxidant challenge establishes the duration of residence in a treated human pocket. Rapid erosion could release the payload before adequate local exposure is achieved, whereas prolonged persistence could extend exposure after the intended treatment phase. The desired degradation profile must therefore be defined against antimicrobial delivery, inflammatory resolution, or defect repair rather than assumed from the term hydrogel.

During validation, drug release should be measured together with dry-mass loss, mechanical integrity, soluble-fragment composition, and local retention under fluid exchange. Polymer labeling can help distinguish a retained network from released cargo, provided that label dissociation is controlled. Tests should include patient-derived fluid and biologically relevant enzymes, buffering, and ionic conditions, followed by in vivo assessment of residual material and tissue response. A reduction in protease or oxidant activity during successful treatment could slow subsequent cleavage, so degradation should also be examined under declining inputs. These studies would establish whether a responsive depot completes the intended dosing period and is subsequently cleared without assuming that therapeutic efficacy proves material resorption.

## 3. Periodontal Pocket Exposure

The periodontal pocket is not a homogeneous reservoir. A subgingival gel may contact the tooth or root surface, residual biofilm, pocket epithelium, and GCF; in a regenerative defect it may also contact blood, exposed connective tissue, and mineralized surfaces. Each interface presents a different candidate input. Host proteases and soluble inflammatory mediators are sampled mainly from GCF, whereas gingipains, volatile sulfur compounds, and short-chain fatty acids (SCFA) arise largely within the subgingival microbial compartment. Oxygen reflects the pocket atmosphere together with the tissue-fluid boundary. Much of the ROS literature, by contrast, concerns intracellular production or downstream molecular damage rather than freely diffusible oxidant in the pocket lumen. A measurement in one domain therefore does not establish the magnitude, duration, or accessibility of the signal at another gel interface.

Dental plaque is a structured polymicrobial biofilm embedded in a hydrated extracellular matrix. Human subgingival specimens show tooth-associated *Actinomyces*, intermediate communities containing *Fusobacterium nucleatum* and *Tannerella* species, and loosely organized outer regions containing spirochetes; *Porphyromonas gingivalis* and *Prevotella* species also occur in microcolonies [23]. This architecture varies among sites and should not be treated as a fixed taxonomic composition. Experimental *F. nucleatum*/*P. gingivalis* biofilms contain extracellular carbohydrates, proteins, and DNA [24]. Human plaque microscopy reveals extracellular strands and membrane-associated vesicles, but morphology alone does not identify every constituent [25]. A fixed bacterial-to-matrix ratio or a predominantly glucan-rich composition should therefore not be assumed for periodontal biofilms.

The interface also contains host material: GCF supplies serum-associated proteins [26], and neutrophil extracellular traps have been demonstrated on the pocket surface and in purulent crevicular exudate [27]. These findings do not define a universal host-DNA fraction in tooth-adherent plaque. For gel design, this mixed microbial and host environment can influence adsorption, enzyme access, and payload transport. Local metabolic production and consumption, diffusion, and GCF exchange are consequently expected to generate interface-dependent exposures, a design inference that requires direct measurement. Periodontal bacteria can also neutralize acidic media through amino-acid metabolism under experimental conditions [28]; residual plaque should not automatically be regarded as an acidic trigger source. Calibration models should specify biofilm maturity and composition, the host-fluid contribution, and the distance between cue production and the responsive network.

GCF sampling illustrates this problem. Strip-collected volume rises from health to periodontitis, but reported mean volumes of 0.04, 0.09, and 0.78 µL represent fluid recovered during a defined interval rather than fixed reservoir volumes [29]. Periotron calibration becomes least reliable below 0.2 µL, which is common at healthy sites [30]. Visually clean strips can contain salivary contamination [31], and repeated sampling can change apparent protein concentration and increase the serum-derived fraction, particularly at inflamed sites [26]. Total recovered amount, concentration, and activity may therefore support different interpretations as fluid volume changes [32,33]. Values derived from eluted strips reflect biological variation together with collection time, recovery volume, evaporation, elution efficiency, and assay performance; they should not be treated as direct measurements of a stable pocket reservoir.

Fluid exchange adds a temporal dimension. GCF flow relates more closely to inflammation than to probing depth, changes after instrumentation, and varies widely among sites and methods [34,35]. These data support dilution and washout as design constraints but do not justify a universal two-minute residence time or another fixed turnover constant calculated by combining volume and flow estimates from separate experiments. A periodontal pocket is not a well-mixed chamber: fluid moves around and through a changing porous gel, while swelling, protein adsorption, payload release, partial degradation, and changes in exposed surface area alter transport over time.

Small-volume flow-through models are more relevant than static sink-release tests. For a commercial chlorhexidine insert, chamber volume, flow rate, and added protease each changed the release profile [36]. A modified system later produced reasonable in vitro-in vivo correlations for three long-acting periodontal products, although exposed surface area disrupted the relation for one formulation [37]. These models do not reproduce a living pocket or validate an endogenous trigger, but they show that flow and exposed surface area materially influence release [36,37]. Hydration, rheological behavior, and bioadhesion are additional material constraints [38], while basal placement, available pocket space, and replacement of drug lost through washout affect clinical retention [39].

Oral fluids are chemically non-equivalent. Paired samples showed that the ionic profile associated with severe periodontitis differed between GCF and saliva [40]. Studies comparing salivary and pocket pH reported both association and no association [41,42]. Recent salivary Tyro3, Axl, and Mer receptor tyrosine kinase pathway biomarkers discriminated periodontal states, but the matrix was whole saliva rather than site-specific pocket fluid [43]. Likewise, heterogeneous blood and salivary biomarker associations in chronic kidney disease do not define causal direction or local material exposure [44]. Such studies can identify pathways for investigation, but oral-rinse active matrix metalloproteinase-8 (aMMP-8) cutoffs, salivary oxidative-damage markers, and blood glucose values should not be used as numerical pocket inputs unless their relation to the gel-contacting compartment is demonstrated.

Even within the same compartment, measured abundance may not equal a material-accessible input. Total MMP-8 antigen includes latent, fragmented, and inhibitor-bound forms, whereas a peptide-crosslinked gel responds only to active enzyme that reaches its substrate. Glutathione and total antioxidant capacity describe buffering rather than H_2_O_2_ concentration, and a diagnostic cytokine threshold does not establish sufficient abundance or transduction. Activity, flux, cumulative exposure, and accessibility may therefore matter more than concentration alone.

**Figure 1:**
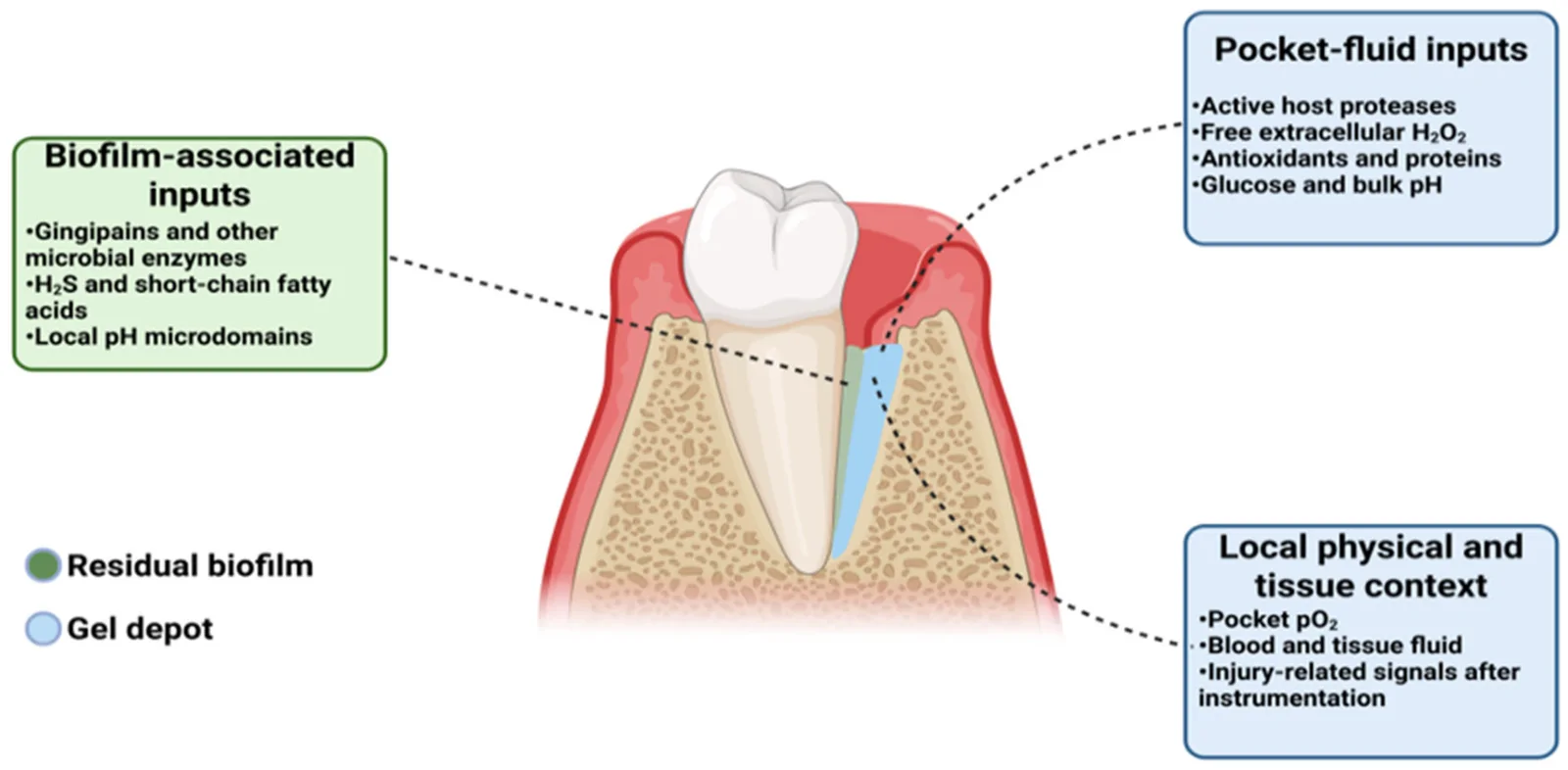
Biological inputs encountered by a subgingival gel within the periodontal pocket. The gel depot may interact with residual biofilm, gingival crevicular fluid (GCF), pocket epithelium, and, depending on the clinical indication and timing of placement, blood and adjacent tissue. Biofilm-associated inputs include gingipains and other microbial enzymes, hydrogen sulfide (H_2_S), short-chain fatty acids, and local pH microdomains. Pocket-fluid inputs include active host proteases, extracellular hydrogen peroxide (H_2_O_2_), antioxidants and proteins, glucose, and bulk pH. Local physical and tissue factors include pocket oxygen tension (pO_2_), blood and tissue fluid, and injury-related signals following periodontal instrumentation. Continuous GCF flow may further modify the magnitude and duration of exposure at each gel interface. Intracellular reactive oxygen species (ROS) should not be considered equivalent to freely diffusible oxidants at the gel surface. Green indicates residual biofilm; blue indicates the gel depot. Created with BioRender.com.

Figure 1 summarizes the biological interfaces and candidate endogenous inputs that a subgingival gel may encounter after placement, highlighting the distinction between biofilm-associated, pocket-fluid, and local tissue-derived signals. The material-relevant form of each candidate cue, representative human periodontal evidence, and corresponding design priorities are summarized in Table 1.

## 4. Post-Instrumentation Trigger Dynamics

Pretreatment disease status does not define the environment at deployment. Subgingival instrumentation is the clinical context in which adjunctive gels are typically placed [2]. The procedure disrupts biofilm and calculus, perturbs the epithelial interface, and changes bleeding and GCF flow [34,68]. MMP-8 concentration may decline during treatment [69], while total antioxidant capacity and glutathione follow different recovery trajectories [70,71]. The post-instrumentation pocket is therefore not simply a weaker version of baseline disease. Calibration based only on cross-sectional pretreatment measurements may misrepresent the signal present at application and during the first hours or days of material residence.

The earliest healing phase may differ qualitatively from both untreated disease and later resolution. One day after nonsurgical treatment of intrabony defects, several cytokines, matrix metalloproteinase-1, matrix metalloproteinase-3, tissue inhibitor of metalloproteinases-1, and basic fibroblast growth factor increased, whereas MMP-8 declined during follow-up [72]. IL-1α and IL-1β also followed different trajectories. A gel placed immediately after instrumentation may therefore respond to tissue injury, transient bleeding, or repair signaling rather than persistent dysbiosis or active destruction. Such responsiveness may still be useful, but the biological state it detects and the intended treatment phase should be stated accurately.

Deployment should be defined clinically rather than collapsed into a generic “periodontitis microenvironment.” Immediate placement after instrumentation, treatment of a recurrent or residual maintenance pocket, and filling of a surgically exposed regenerative defect involve different biofilm burdens, fluids, oxygenation, tissue contacts, trigger trajectories, residence requirements, and therapeutic objectives. A threshold optimized in an untreated ligature model may not operate after ligature removal or debridement. Studies should report ligature status, debridement and irrigation, the interval to gel placement, bleeding at application, and whether the cue was measured at that time.

The material also modifies its own input. Acid- or base-labile groups buffer protons, boronates consume oxidants, and enzyme-cleavable networks adsorb proteins. Swelling and degradation change interfacial area and diffusion. Biological calibration is therefore a dynamic interaction between a changing lesion and a reactive depot, not a single comparison between a pretreatment biomarker value and a chemical threshold.

## 5. Periodontal pH as a Trigger

### 5.1. Human Periodontal pH

Acid-responsive periodontal gels are often justified by describing diseased pockets as pH 5.0–6.5, but direct human evidence does not support this as a general periodontitis range. A methodologically informative in situ study used glass microelectrodes at 184 sites in 69 participants spanning periodontal health to periodontitis and reported a participant-level mean pH of 6.92 ± 0.03, without a significant association between pH and local gingivitis or pocket depth [45]. Bench experiments demonstrated an upward bias of up to 1.5 pH units with antimony electrodes under reducing conditions and a rise of approximately one pH unit after carbon dioxide loss from thin films of saliva or bicarbonate-buffered medium [45]. Although cross-sectional and unable to resolve biofilm microdomains, the study challenges both a universal acidic premise and uncritical reliance on ex vivo alkalinity.

Other human measurements are heterogeneous. A small ion-sensitive field-effect transistor study reported means of 6.35 at diseased and 6.59 at healthy sites [46], while a capillary-fitted microelectrode study reported an overall crevicular-fluid pH range of 7.0–10.2 (mean 8.5 ± 0.7), with significant inverse correlations with pocket depth and gingival inflammation [41]. By contrast, capillary-collected GCF measured ex vivo ranged from 6.90 to 8.66 with increasing inflammation [73]. Under controlled temperature and carbon dioxide tension, inflamed-site GCF had a mean pH of 7.96, rising to 8.38 when carbon dioxide was omitted [74]. Genuine biological variation, sampling position, electrode chemistry, and gas handling therefore remain inseparable from the reported value.

Longitudinal evidence remains sparse. Pocket pH increased after irrigation or scaling and root planing in one study, approaching an unusually high healthy reference near pH 9; the direction may be informative, but the absolute values raise concerns about sensor behavior [75]. Repeated insertion of an iridium-oxide probe changed the second or third reading in nine of ten participants, showing that measurement itself can perturb the environment [76]. Another series reported a mean of 6.7 and a range of 5.8–7.2, with little effect from acid rinsing and inconsistent shifts during isolated inflammatory episodes [42]. A 2025 study reported pH 6.73 ± 0.14 in health and 8.19 ± 0.29 in stage II, grade B periodontitis, but used colorimetric strips in only 15 participants per group [47]. Together, these studies show method-sensitive variability rather than a validated activation threshold.

A reported GCF pH near 5.4 represents a group mean obtained using indicator strips in participants with gingivitis and cervical lesions, rather than a measurement of acidic microdomains in periodontitis pockets [77]. Acidic niches have been proposed as targets for pH-responsive periodontal delivery [78], but the human measurements reviewed here do not establish their spatial extent, persistence, or accessibility to a gel after debridement.

Periodontal microbial metabolism also argues against a single pH direction. Under experimental conditions, *Porphyromonas gingivalis*, *Prevotella intermedia*, and *Fusobacterium nucleatum* can neutralize acidic media through amino acid metabolism and ammonia production [28]. This does not prove alkaline human pockets; it shows that pH depends on substrate availability, community metabolism, buffering, and location.

### 5.2. Acid-Responsive Gel Testing

Acid responsiveness can arise from ionization, covalent-bond hydrolysis, or dissolution of an incorporated carrier. In chitosan-derived gels, protonation of residual amines changes charge, hydration, and polymer association without necessarily cleaving the backbone. The naringin thermogel combined amphiphilic carboxymethyl-hexanoyl chitosan, β-glycerophosphate, and glycerol; its temperature-dependent assembly and pH-dependent release are therefore distinct material behaviors [79]. In the embelin formulation, aldehydes on oxidized dextran react with primary amines on carboxymethyl chitosan to form dynamic imine crosslinks, linking the network chemistry to injectability and self-repair [17]. By comparison, the polyglycerol nanogel incorporates acid-cleavable acetal groups within its architecture and triclosan linkage, allowing acidic exposure to promote both de-crosslinking and liberation of chemically unmodified triclosan [80]. These mechanisms need not share an activation range or reversibility. Moreover, pH-dependent payload solubility and partitioning can alter apparent release independently of matrix breakdown, making paired measurements of network structure and drug speciation necessary.

Chitosan-based systems carrying an anti-glycation agent or naringin released payload more rapidly at pH 5.5–6.5 than at pH 7.4 and improved experimental periodontitis outcomes [79,81]. In the reports examined, neither study documented local pocket pH in vivo, tested the complete gel in native GCF under flow, or included a chemistry-matched nonresponsive comparator with comparable baseline release. These experiments establish pH-dependent release and support the composite formulations, but do not verify acidic triggering or its contribution in vivo.

Newer systems show the same attribution problem. An embelin-loaded dynamic hydrogel was compared at pH 5.0 and 7.4, with an early release difference of 15% versus 10% at 8 h before cumulative release approached 90% by day 15 [17]. That study also reported lower periodontal-fluid pH in diseased than healthy rats, but did not show that this difference governed release in vivo [17]. A zeolitic imidazolate framework-8 (ZIF-8)/metronidazole gel released 70%, 58%, and 47% at pH 5.0, 6.5, and 7.5, respectively, by 60 h, indicating a clear gradient but substantial off-state release [82]. A polyglycerol nanogel carrying triclosan and deferoxamine showed strong acetal cleavage below pH 6 and improved a ligature-induced mouse model, but pH below 6 was not demonstrated at the implanted site [80]. These systems may be useful sustained-release formulations, but endogenous acidic activation remains unverified.

### 5.3. Alkaline-Responsive Gel Testing

A 2025 alkaline-responsive poly(ethylene glycol) (PEG) hydrogel challenged the conventional acidic-inflammation premise. The thioacetate-containing gel was designed for alkaline-responsive rutin delivery. Rutin release was tested in phosphate buffer at pH 6.6–8.2, and the formulation showed anti-inflammatory and tissue-protective effects in cell and mouse experiments [14]. The design is compatible with reports of alkaline GCF, but local pocket pH could not be verified from the accessible reporting, and the human evidence remains method-dependent. Acidic and alkaline designs should therefore be treated as competing engineering hypotheses applied to a spatially heterogeneous signal rather than as successive corrections from an incorrect to a correct premise.

For pH to act as a disease-selective switch, the responsive element must sample the relevant interface. A nanoparticle near residual biofilm may experience a different microdomain from a bulk network spanning pocket fluid, and debridement may remove the niche used to justify the design while leaving residual apical biofilm outside the main gel volume. Carbon dioxide exchange, protein buffering, salivary ingress, bleeding, and the gel’s own ionizable groups further alter local pH during release. Validation should map gel–biofilm and gel–epithelium interfaces before and after instrumentation and test response across the observed near-neutral region, not only at two distant buffer conditions. Current evidence supports pH-sensitive chemistry, not a universal periodontal activation window.

On the available human evidence, we consider calibrated glass microelectrodes used directly in situ the best-supported reference approach for quantitative pocket-fluid pH measurement. This is a methodological recommendation, not a formally established clinical gold standard. Glass electrodes avoid the marked redox-related bias demonstrated with metallic antimony electrodes, while direct measurement reduces artefacts from fluid collection and carbon dioxide loss [45]. Calibration across the expected range, temperature control or compensation, tip position, stabilization time, and minimal pocket disturbance should be documented. Repeated insertion can change subsequent readings and should not be treated as independent replication [76]. Glass-tip fragility and restricted access remain practical limitations, and a point measurement cannot map every gel–biofilm microdomain. Miniaturized solid-state probes should be validated under protein-rich, reducing, bicarbonate-buffered conditions before their readings are used to define a narrow release threshold. Colorimetric strips and ex vivo samples are less suitable for this purpose when direct quantitative measurement is feasible. The priority is therefore standardized in situ measurement at the intended deployment site, with uncertainty and treatment timing reported explicitly.

## 6. Protease-Responsive Gels

### 6.1. MMP-8 as a Trigger

Protease-responsive networks offer catalytic amplification: one active enzyme can cleave multiple crosslinks or release motifs, potentially producing a material response at lower concentrations than a stoichiometric bulk transition. MMP-8 is particularly relevant because neutrophil-derived collagenolysis is closely linked to periodontal tissue breakdown [83]. However, “MMP-8 level” can denote total antigen, active immunoreactivity, processed fragments, inhibitor-bound enzyme, or catalytic activity. ELISA and immunofluorometric methods recognize different molecular pools, whereas activity assays depend on the substrate and assay environment. A peptide-crosslinked gel responds only to enzyme that remains active, enters the network, and accesses its sterically constrained substrate.

Human GCF studies do not provide a single transferable trigger concentration. In a full-mouth analysis of nine women with generalized chronic periodontitis, patient-level mean aMMP-8 immunoreactivity ranged from 3.2 to 23.7 ng/mL and related only weakly to probing depth despite dense site sampling [48]. A separate ELISA reported mean total MMP-8 of 7.05 ng/mL in health, 56.97 ng/mL in nonsmoking periodontitis, and 68.96 ng/mL in smoking periodontitis [50]. These are not conflicting estimates of the same entity. Different laboratory and chairside assays applied to the same GCF samples also produced markedly different values and disease separation [49]. A systematic review found baseline MMP-8 values spanning several orders of magnitude across only five studies and no reliable site-specific threshold [84]. Much of the spread reflects sampling, elution, normalization, and assay recognition rather than a physiological distribution.

Diagnostic performance does not resolve analyte equivalence. A meta-analysis estimated median sensitivity of 76.7% and specificity of 92.0% for GCF MMP-8 in periodontitis detection, but the included studies used heterogeneous assays and diagnostic classifications rather than direct crosslink-cleavage measurements [85]. Chairside test results and measured MMP-8 levels changed after scaling and root planing at a fixed immunological cutoff [86], but that cutoff is not a catalytic threshold for a gel substrate. MMP-8 diagnostic performance differed between immunofluorometric and ELISA platforms [87]. Longitudinal response patterns and optimal cutoffs also differed by smoking status [88]. These findings support biomarker utility, not transfer of an immunological cutoff to a gel-cleavage threshold.

Longitudinal studies strengthen the biological relevance of MMP-8 while weakening the case for a universal switch. During six months of untreated monitoring, pooled baseline GCF MMP-8 antigen was modestly higher in patients who later developed attachment loss, but distributions overlapped and selected sites were pooled at patient level [51]. A larger bimonthly study in systemically healthy nonsmokers found consistently higher MMP-8 at progressing than stable sites, whereas matrix metalloproteinase-9 (MMP-9) was higher at stable sites [52]. Differences in population, smoking, diabetes, sampling, and follow-up may explain part of the divergence. These findings support association with progression, not an on/off material threshold, and show that related proteases can follow distinct or even opposing trajectories.

### 6.2. Recombinant MMP-8 and Native GCF

A frequently cited PEG hydrogel used the matrix metalloproteinase-cleavable peptide CGPQGIWGQC and carried minocycline, bovine serum albumin, or an antibacterial peptide [19]. Degradation and release were tested with 10 or 20 nM activated recombinant human MMP-8; because activity declined, the enzyme solution was renewed every 48 h. Patient aMMP-8 values reported as immunoreactive mass cannot be converted into an equivalent catalytic exposure. Purified enzyme activity, assay recognition, exposure renewal, inhibition, diffusion, network accessibility, and substrate kinetics differ fundamentally. The experiment therefore demonstrates responsiveness to sustained recombinant enzyme but does not establish a human activation threshold or a quantitative mismatch that can be expressed as a valid fold difference. Native GCF and a periodontal in vivo model were not tested, and a later corrigendum did not change the reported conclusions [19].

Native-fluid testing would address more than dose reduction. MMP-8 occurs in latent, active, inhibitor-associated, and processed high-activity forms [83]. Tissue inhibitors of metalloproteinases and competing protease activities contribute to the native GCF cleavage environment [89,90]. Protein adsorption, ionic conditions, and crosslink accessibility are material variables that should be tested directly rather than inferred from enzyme abundance. Peptide selectivity should be challenged against MMP-9, neutrophil elastase, gingipains, and other pocket proteases at concurrent activities. Selective inhibitors and experimentally verified cleavage-resistant peptide controls can help distinguish intended cleavage from nonspecific swelling, adsorption, or cross-cleavage. No native-GCF input–output curve was reported for this gel.

Human functional studies likewise show that active and activatable collagenase activity, matrix metalloproteinase immunoreactivity, tissue inhibitor of metalloproteinases abundance, and multienzyme panels are not interchangeable [89]. In a small pilot, a panel combining active MMP-8, elastase, and sialidase classified internally defined treatment outcomes better than selected individual activities, but no externally validated biochemical threshold was established [90]. Such panels may improve biological characterization without providing a preset challenge concentration for a gel.

### 6.3. Elastase and MMP-9

Neutrophil elastase is associated with periodontal inflammation and progression, but most studies use substrate-dependent activity assays. A chairside elastase score predicted combined bone and attachment loss with 82% sensitivity and 66% specificity [91], whereas experimental gingivitis increased median fluorogenic elastase activity threefold [53], showing that the signal is not specific to destructive periodontitis. After therapy, total and free elastase activity, MMP-8, and collagenolytic activity declined, although their residual post-treatment patterns differed [92,93]. Because the assays quantify different molecular entities, their trajectories should not be treated as interchangeable. Activity against a small substrate does not ensure entry into a gel or cleavage of a sterically constrained crosslink. Elastase and MMP-9 are therefore valuable specificity challenges, not validated universal triggers.

### 6.4. Gingipain-Responsive Gels

Bacterial proteases could make release more dependent on pathogenic biofilm than on host inflammation. In severe chronic periodontitis, pocket-wash arginine-specific gingipain reached 1.5 µM and correlated with *P. gingivalis* abundance, whereas paper strips recovered much less enzyme [55]. This sampling dependence matters because a gel occupying the lumen may encounter enzyme pools missed by a strip. Lysine-specific gingipain was detected in only 13 of 77 *P. gingivalis*-positive sites, and cleaved IgG1 occurred in some samples without detectable lysine-specific gingipain [54]. Sampling method, protease identity, strain abundance, substrate specificity, and native inhibition therefore determine whether a site presents an accessible signal.

A thermosensitive PEG hydrogel incorporated an arginine-specific gingipain A (RgpA)-cleavable module and stromal cell-derived factor 1 and was tested with 1 or 10 nM recombinant RgpA in a *P. gingivalis*-dependent rat model [15]. This provides a coherent pathogen–substrate relation and in vivo therapeutic evidence, but not selective cleavage by patient-derived fluid. Human pocket-wash values and recombinant RgpA challenges differ in enzyme preparation, sampling, and activity state and cannot define a calibrated population threshold. The system may be appropriate for *P. gingivalis*-rich sites rather than universally responsive to periodontitis; that is a stratification property, not necessarily a design failure.

Pathogen-specific triggering must also account for ecology. In an experimental model, low-abundance *P. gingivalis* remodeled a commensal community and promoted complement-dependent inflammatory bone loss without becoming numerically dominant [94]. Gingipain activity may therefore identify one mechanistically important ecology, but a weak signal does not imply biological inactivity at a polymicrobial site. Trigger selectivity and patient coverage are distinct design properties.

Proteolysis remains the strongest amplified mechanism examined. MMP-8 has the broadest human biomarker base, gingipains offer greater microbial specificity in a subset of sites, and MMP-9 and elastase are useful competing inputs. Yet no candidate has a human pocket activity distribution that can be overlaid with a complete native-fluid gel-cleavage curve.

## 7. Periodontal Redox Cues

### 7.1. Human Redox Measurements

Oxidative stress is integral to periodontal inflammation, but an H_2_O_2_ bolus represents only one component of a dynamic redox environment. Human studies more often measure malondialdehyde, 8-hydroxy-2′-deoxyguanosine, protein oxidation, total oxidant status, superoxide dismutase, or antioxidant capacity. These endpoints reflect accumulated damage or aggregate buffering and cannot be converted into free H_2_O_2_ at the gel surface. Intracellular ROS may be high while extracellular fluid remains strongly buffered. Conversely, a brief interfacial flux may be biologically important without producing a stable concentration recoverable on a strip.

A systematic review and meta-analysis found higher GCF malondialdehyde in periodontitis, whereas total oxidant status and superoxide dismutase did not differ consistently; heterogeneity across 32 studies was high [95]. These endpoints describe different redox constructs and cannot be pooled into a free-ROS concentration. They support oxidative involvement in periodontal pathology without defining the magnitude or duration of the material input.

A 2024 pilot directly quantified H_2_O_2_ in strip-collected, frozen GCF using an Amplex Red-peroxidase assay and reported means of 5.55 ± 0.56, 7.76 ± 0.93, and 11.19 ± 1.57 µM in health, gingivitis, and periodontitis, respectively [56]. The groups included only 11 controls, eight participants with gingivitis, and 12 with periodontitis. Peroxide instability, freeze–thaw handling, matrix effects, and unclear site-level aggregation limit interpretation. The study provides an order-of-magnitude bulk-fluid reference, not a validated activation threshold or an estimate of transient interfacial peaks.

Human GCF also contains substantial antioxidant capacity. Mean reduced glutathione (GSH) concentrations were 1.90 mM in health and 1.18 mM in periodontitis, while mean total antioxidant capacity in the same study was 1.35 and 0.66 mM Trolox equivalents, respectively [57]. These measures are not stoichiometric H_2_O_2_ equivalents, but they show that peroxide is encountered in buffered fluid rather than inert solution. Total antioxidant capacity increased after successful nonsurgical therapy in one cohort, whereas glutathione and oxidized glutathione measures remained abnormal in another [70,71]. The periodontal redox state is therefore neither a simple peroxide concentration nor a single analyte with a uniform trajectory.

### 7.2. H_2_O_2_ Challenge Conditions

Representative phenylboronate-based periodontal gels were characterized with 100 µM to 1 mM H_2_O_2_. A poly(vinyl alcohol) (PVA) hydrogel crosslinked with a ROS-cleavable bis-boronic acid linker carrying macrophages and a C5a receptor antagonist lost integrity rapidly at 1 mM and disappeared by 120 h at 100 µM [22]. A hyaluronic acid/PVA gel co-delivering minocycline and an iron-quercetin nanoparticle used 0, 100, and 1000 µM in degradation and release studies, while cellular stress models used 100–300 µM [18]. A calcitonin gene-related peptide-loaded PVA/boronate gel was tested at 1 mM and underwent near-complete degradation over 15 days [96]. These conditions exceed the limited reported bulk-GCF estimate and generally omit physiological antioxidant buffering. That comparison indicates uncertainty rather than a validated fold mismatch, because the pilot did not resolve transient peaks, flux, or interfacial microdomains.

Efficacy also cannot automatically be attributed to ROS triggering. The cited systems combine responsive degradation with payload action, macrophage delivery, antioxidant nanoparticles, and changes in gel mechanics. In the calcitonin gene-related peptide study, the blank responsive gel did not significantly improve the in vivo outcome, and the authors suggested that persistent diabetic oxidative stress may have exceeded the material’s scavenging capacity [96]. Without controls that isolate ROS-dependent material behavior, the contribution of the trigger-sensitive bond remains unresolved even when the complete formulation improves inflammation or bone outcomes.

### 7.3. Triggered Release and ROS Scavenging

Boronate oxidation can both cleave a crosslink and consume oxidant. As degradation proceeds, the material may reduce the input that accelerates release, creating a potentially useful but self-limiting response. A single cumulative-release curve in a large static bath cannot resolve this behavior. The relevant mass balance includes oxidant production and diffusion, antioxidant buffering, crosslink density, gel volume, GCF exchange, payload potency, and the changing interfacial area of the degrading depot.

The distinction is especially important when antimicrobial ROS generation is combined with later antioxidant or regenerative functions, because these aims require opposing oxidative exposures at different spatial and temporal scales. Photodynamic or chemodynamic systems seek local oxidative killing, whereas ROS-labile or scavenging gels aim to reduce host injury. Calibration would require measurement of the relevant oxidant at the gel interface, continuous low-rate challenge in artificial GCF containing proteins and antioxidants, and confirmation in patient-derived fluid with catalase or scavenger controls. Current evidence establishes in vitro H_2_O_2_ responsiveness and selected preclinical therapeutic activity, not calibration to human periodontal oxidant exposure.

## 8. Oxygen and Microbial Metabolites

### 8.1. Oxygen Tension and Oxygen-Releasing Gels

The partial pressure of oxygen (pO_2_) is one of the few physical variables measured directly at the base of untreated human pockets. Across 111 pockets 5–10 mm deep, values ranged from 5 to 27 mmHg, with a mean of 13.3 mmHg; deeper pockets had lower mean pO_2_ than 5–6 mm pockets [58]. A related study associated lower pO_2_ with a greater proportion of spirochetes and higher pO_2_ with more *Capnocytophaga* [97]. These historical data support a depth-related gradient in advanced untreated disease, but they derive from small culture-era cohorts and do not distinguish pocket anatomy, microbial consumption, tissue perfusion, and active destruction.

Later data linked pocket pO_2_ to probing depth and gingival oxygen saturation but not consistently to gingival inflammation, plaque, or GCF flow [98]. Oxygen is therefore a measurable local condition rather than a validated marker of active disease. A true oxygen-sensing material should state whether it targets deep-pocket anatomy, microbial metabolism, tissue perfusion, or a combination of these states.

Several gels address hypoxia by releasing oxygen rather than sensing pO_2_. A calcium peroxide/gelatin methacrylate system produced an oxygen burst and reduced mono- and multispecies biofilms in vitro [99]. A MgO_2_/polydopamine thermosensitive gel released oxygen and magnesium ions under acidic conditions and improved periodontal outcomes in rats [100]. These may be useful oxygenating therapies, but output was governed by hydration, acidity, or formulation kinetics rather than feedback from local pO_2_. The cited oxygen-generating systems did not directly couple release to measured local oxygen tension. Oxygen-generating therapy should therefore not be equated with hypoxia-responsive delivery.

### 8.2. Hydrogen Sulfide

Headspace gas chromatography of acidified gingival-fluid samples from 33 participants detected H_2_S in 61 of 79 sites, with a maximum of 1.9 mM; methyl mercaptan was less frequently detected and reached 0.16 mM [59]. Individual site concentrations were plotted against probing depth, but these measurements do not establish free H_2_S exposure at native pocket pH or a representative population cutoff. Several oral taxa produce these volatile sulfur compounds, so the signal reflects community metabolism rather than a unique pathogen [101]. Because the pocket study reported maxima rather than distributions, predates current classification, and was sensitive to volatility and handling, 1.9 mM cannot serve as a clinical cutoff or a representative population exposure.

A 2026 hydrogel used H_2_S-mediated azide-to-amine conversion to increase antibacterial activity while sustaining oxygen release [102]. At 1 mM H_2_S, conversion reached 76% by 15 h and 84% at the later endpoint; final conversion rose to 86% and 92% at 1.5 and 2.0 mM, respectively. These nominal concentrations overlap the historical maximum, but equivalence between the acidified human measurements and the material-active sulfide input has not been established. Patient-derived fluid was not tested, and local H_2_S was not measured in the rat model; biological calibration therefore remains unverified. A separate redox-active hydrogel released H_2_S as therapy rather than using endogenous H_2_S as a trigger [103].

A zinc oxide/hyaluronan gel instead consumed H_2_S and reduced animal-pocket values from 0.7–0.8 to 0.3–0.4 mM while improving inflammatory and bone outcomes [104]. This is a useful example of measuring an endogenous metabolite during treatment, but scavenging remains distinct from trigger transduction and the relevant range may depend on the disease model.

### 8.3. SCFAs

Subgingival fermentation products may provide more stable microbial cues than ROS. Historical measurements of acid-extracted subgingival plaque reported mean propionate and butyrate values of 9.5 ± 1.8 and 2.6 ± 0.4 mM in severe disease, compared with 0.8 ± 0.3 and 0.2 ± 0.04 mM in mild disease [60]. These measurements support a disease association but do not establish native-GCF concentrations or dissolved exposure at the gel surface. We did not identify a directly SCFA-responsive periodontal gel among the studies examined, which contrasts with the limited human quantification available for several more frequently engineered cues.

More recent data reported median GCF propionate of 10.23 versus 2.71 mM and butyrate of 2.63 versus 0.00 mM in advanced periodontitis and health, with higher values in deep than shallow pockets but no distinction between grades B and C [61]. Longitudinal studies also found treatment-related changes: volatile fatty acids declined after nonsurgical therapy, reached low values within weeks, and later tended to rise again [105,106]. SCFAs therefore have comparatively strong same-compartment concentration and treatment-response evidence. Neither longitudinal study linked its measured concentrations to site progression or to a material-response threshold.

### 8.4. Cytokines and Endotoxin

Cytokines illustrate the difference between a biomarker and a practical trigger. An older GCF study reported IL-1β ranges of 22.8–150 ng/mL in health and 85.8–882.2 ng/mL in periodontitis, with substantial overlap [65]. A later multiplex study identified diagnostic thresholds in the low-ng/mL range and showed that smoking altered the optimal cutoffs [66]. The order-of-magnitude difference cannot be interpreted without considering assay, sampling, case definition, normalization, and biological variation. A noncatalytic cytokine is unlikely to alter bulk gel chemistry without an affinity-, antibody-, or aptamer-based transduction element.

In one small clinical study, GCF endotoxin was higher in generalized aggressive periodontitis than in healthy controls and declined after surgery, but the study did not establish a population distribution suitable for setting a material threshold [67]. *P. gingivalis* LPS is also structurally heterogeneous, and different lipid A forms produce different cellular responses [107]. In many gel studies, LPS is used to stimulate macrophages or periodontal cells and the material is judged by its ability to reduce downstream cytokines; LPS stimulates the cells rather than the gel. Cytokines and endotoxin are therefore better treated as biological readouts or future molecular-recognition inputs than as mature autonomous switches.

## 9. Glucose-Responsive Gels in Diabetic Periodontitis

Glucose responsiveness is often presented as precision therapy for diabetes-associated periodontitis. The bidirectional clinical association, altered inflammatory and healing responses, and the role of glycemic control in periodontal grading are well established [1,108,109], but blood glucose, post-probing crevicular blood, and native GCF are different exposure matrices. GCF glucose ranged from 0.01 to 0.50 mM in ten healthy adults studied before and after meals [62]. A separate study of ten adults with type 2 diabetes reported 12.8–278 mg/dL (0.71–15.4 mM), using a 200-nL collector and enzymatic fluorometry [63]. Another microassay reported GCF-to-blood ratios of 1:2 in periodontitis and 1:9 in health [64]. These study-specific measurements permit provisional numerical comparisons but do not establish glucose exposure in clinically characterized diabetic periodontal pockets or after instrumentation.

Systemic glycemic status can alter local inflammation and microbial composition without acting through a single GCF glucose concentration. In participants with type 2 diabetes and periodontitis, poorer control was associated with higher GCF IL-1β after adjustment for clinical variables [110]. A small sequencing study also found differences in subgingival community composition between adequately and inadequately controlled diabetes, including fermentative taxa [111]. These findings support a phenotype-specific environment containing several correlated modifiers rather than a single glucose threshold.

Gingival crevicular blood collected after probing can correlate with finger-stick glucose, but that evidence concerns diabetes screening rather than native gel exposure, and agreement worsens when little bleeding is produced [112]. A gel placed after instrumentation may briefly contact blood, yet this iatrogenic exposure differs from native GCF glucose. A material activated mainly by postprocedural bleeding would be wound-responsive rather than specifically diabetes- or periodontitis-responsive.

A glucose-responsive core–shell platform comprised a ZIF-8 core containing minocycline, GOx, and catalase, surrounded by a crosslinked calcium–alginate hydrogel shell [113]. GOx converted glucose to gluconic acid, dissolving ZIF-8 and increasing drug release and biofilm penetration. At 5, 15, and 35 mM glucose, 25%, >50%, and >75% of minocycline was released within 90 min. The 5 and 15 mM challenges overlap the range reported in the small human type 2 diabetes series [63], whereas 35 mM exceeds that range. This supports provisional numerical correspondence, but the rat model measured systemic rather than local glucose, and biofilm acidity could dissolve ZIF-8 independently of glucose.

Newer systems designed for diabetic periodontitis or diabetic alveolar bone defects combine glucose with acidic pH and peroxide or use hierarchical GOx/catalase designs [16,114,115]. A cationic phenylboronic acid hydrogel was tested separately with 11.1 mM glucose, pH 5.5, and peroxide [114], whereas another system showed accelerated degradation under combined inputs [16]. A hierarchical GOx/catalase construct improved regeneration in a diabetic rat alveolar bone-defect model [115]. These studies demonstrate responsive chemistry and therapeutic activity, but none measured native GCF glucose at the treated site, defined the combined pocket exposure, or established phenotype-specific release.

GOx systems consume oxygen, generate H_2_O_2_, and lower pH through gluconic acid; catalase may recycle peroxide to oxygen, but reaction rate and spatial accessibility determine the net effect. Behavior can therefore vary with pocket depth, residual biofilm, oxygenation, buffering, and enzyme localization even at the same glucose concentration. Because net behavior depends on local oxygenation and buffering, a systemic glycemic label cannot substitute for measurement of the pocket activation threshold.

## 10. Multistimulus Systems and Selectivity

Multistimulus systems are often presented as more selective, but selectivity depends on response logic rather than the number of responsive bonds. If pH, ROS, and glucose each accelerate degradation independently, additional inputs may broaden activation. Demonstrating AND-type behavior requires a concentration matrix and both single-input controls. The cited studies either tested the inputs separately or demonstrated faster degradation under concurrent glucose and peroxide challenges [16,114]; neither established whether any single input was already sufficient to produce the claimed therapeutic release in vivo.

Biological inputs are chemically coupled. GOx converts glucose into acid and peroxide, boronate oxidation changes crosslink density, and pH alters enzyme activity and ZIF-8 dissolution. A nominally dual-responsive gel may therefore contain one upstream cue and its reaction products rather than two independent disease signals.

Sequential antimicrobial, anti-inflammatory, and regenerative delivery is plausible, but separate release curves or a late bone endpoint does not establish stage-specific release. Studies should use multidimensional input matrices, matched single-responsive networks, longitudinal measurement of both cues and payloads, and evidence that the sequence persists under realistic post-instrumentation trajectories. Otherwise, “multiresponsive” describes chemistry rather than greater periodontal precision.

Box 1 defines the biological calibration categories used in this review, and Table 2 summarizes the experimental challenge conditions, material responses, and corresponding calibration assessments for representative responsive periodontal gels.

Box 1 **Operational definitions of biological calibration categories.**

Unresolved—undefined human exposure. Quantitative exposure to an essential cue is undefined in the intended human compartment and application, preventing comparison with the material operating range.

Unverified—uncertain input equivalence. Human measurements exist, but assay meaning, molecular form, bioavailability, or turnover prevents reliable translation into the material-active input.

Limited—unsupported challenge or application premise. The cue permits comparison, but human evidence inadequately supports the response-driving condition or intended application. Overlap at other test conditions does not resolve this gap.

Partial—provisional exposure correspondence. Human measurements overlap material testing of the same nominal cue, but exposure characterization, native-fluid validation, or in vivo cue-response attribution remains incomplete. Historical maxima provide only provisional support. Precursor-only responses are designated Partial, component-level only, without establishing whole-gel activation.

Not assessable—insufficient accessible information. Essential challenge or response data could not be verified from accessible sources; this does not imply that the original study omitted them.

**Table 2:** Representative responsive periodontal gels: material test conditions, material responses, and human evidence.

Responsive Gel	Trigger	Material Test Conditions	Material Response	Biological Calibration: Category and Rationale
PTB-chitosan hydrogel [81]	Acidic pH	pH 5.5, 6.5 and 7.4	Marked acceleration at pH 5.5; 68.14% versus 63.19% release at pH 6.5 versus 7.4 after 288 h	Partial: response at pH 6.5 overlaps selected human measurements, but discrimination from pH 7.4 was modest
Naringin thermogel [79]	Acidic pH	pH 5.5, 6.5 versus 7.4	Faster release under acidic conditions, including pH 6.5	Partial: response at pH 6.5 overlaps selected human measurements; local pH-dependent activation remains unverified
Embelin dynamic hydrogel [17]	Acidic pH	pH 5.0 versus 7.4	15% vs. 10% release at 8 h; 90% by day 15	Limited: the pH 5.0 challenge is not adequately supported by relevant human pocket/GCF measurements
ZIF-8/metronidazole hydrogel [82]	Acidic pH	pH 5.0, 6.5 and 7.5	70%, 58%, and 47% release at 60 h	Partial: response at pH 6.5 overlaps selected human measurements; pH 5.0 is poorly supported
Triclosan/deferoxamine polyglycerol nanogel [80]	Acidic pH	pH 5.5 versus 7.4; acetal cleavage below pH 6	At pH 5.5: 78% triclosan and ≈72% deferoxamine at 24 h; triclosan ≈ 6% at pH 7.4	Limited: pH < 6 not verified locally in vivo
Rutin-PEG gel [14]	Alkaline pH	pH 6.6–8.2	Matrix integrity decreased and rutin release increased with pH	Partial: response conditions overlapped with reported alkaline GCF values, but in vivo pH remained uncertain.
MMP-8-PEG gel [19]	MMP-8	10 and 20 nM activated recombinant enzyme	Network degradation and payload release	Unverified: recombinant activity is not equivalent to human immunoreactivity
RgpA thermogel [15]	RgpA	1 and 10 nM recombinant enzyme	Cleavage-linked stromal cell-derived factor-1 release	Unverified: human pocket-wash Rgp measurements do not establish equivalent RgpA-mediated cleavage of this network
PVA-TSPBA hydrogel [22]	H_2_O_2_	100 µM and 1 mM H_2_O_2_	H_2_O_2_-dependent degradation and cell release	Limited: H_2_O_2_ challenge exceeds direct GCF estimate; antioxidant buffering absent
HA/PVA boronate hydrogel [18]	H_2_O_2_	0, 100 and 1000 µM H_2_O_2_	H_2_O_2_-triggered degradation and co-release	Limited: H_2_O_2_ challenge exceeds direct GCF estimate; antioxidant buffering absent
CGRP-PVA/boronate hydrogel [96]	H_2_O_2_	1 mM H_2_O_2_	Near-complete degradation by day 15	Limited: H_2_O_2_ challenge exceeds direct GCF estimate; antioxidant buffering absent
H_2_S-responsive nanoparticle hydrogel [102]	H_2_S	0.01–2.0 mM H_2_S	84–92% conversion at 1–2 mM H_2_S; antibacterial activity increased	Unverified: nominal overlap with historical sulfide measurements; equivalence to the active material remains unconfirmed
CaAlg hydrogel-coated GOx/CAT/ZIF-8 system [113]	Glucose	5, 15 and 35 mM glucose	At 90 min: 25%, >50%, and >75% minocycline release	Partial: 5 and 15 mM overlap reported diabetic GCF values; 35 mM exceeds that range, and local triggering remains unverified
PBA cationic dynamic hydrogel [114]	Glucose + H_2_O_2_ + acidic pH	11.1 mM glucose, pH 5.5 and 0.1% H_2_O_2_, tested separately	Faster degradation/release; 0.1% H_2_O_2_ caused complete degradation within 20 h	Partial for glucose; Limited for pH/H_2_O_2_: 11.1 mM overlaps reported diabetic GCF values; diseased-pocket exposure and pH/H_2_O_2_ challenges remain insufficiently supported
Glucose/ROS/pH-responsive hydrogel [16]	Glucose + H_2_O_2_ + acidic pH	Conditions could not be verified	Accelerated degradation and minocycline/GSK2606414 release reported	Not assessable: insufficient verified information

Abbreviations: CAT, catalase; CGRP, calcitonin gene-related peptide; GCF, gingival crevicular fluid; GOx, glucose oxidase; HA, hyaluronic acid; MMP-8, matrix metalloproteinase-8; PBA, phenylboronic acid; PEG, poly(ethylene glycol); PTB, N-phenacylthiazolium bromide; PVA, poly(vinyl alcohol); Rgp, arginine-specific gingipain; RgpA, arginine-specific gingipain A; ROS, reactive oxygen species; ZIF-8, zeolitic imidazolate framework-8. Note: Limited, Partial, Unverified, Unresolved, and Not assessable are descriptive calibration categories used to summarize the correspondence between material test conditions and available human periodontal evidence. They do not constitute a validated scoring system or ordinal ranking and should not be interpreted as measures of study quality or therapeutic efficacy. A simplified application of the reporting and validation checklist in Section 11 to each system is provided in Table S2.

## 11. Requirements for Biological Calibration

**Figure 2:**
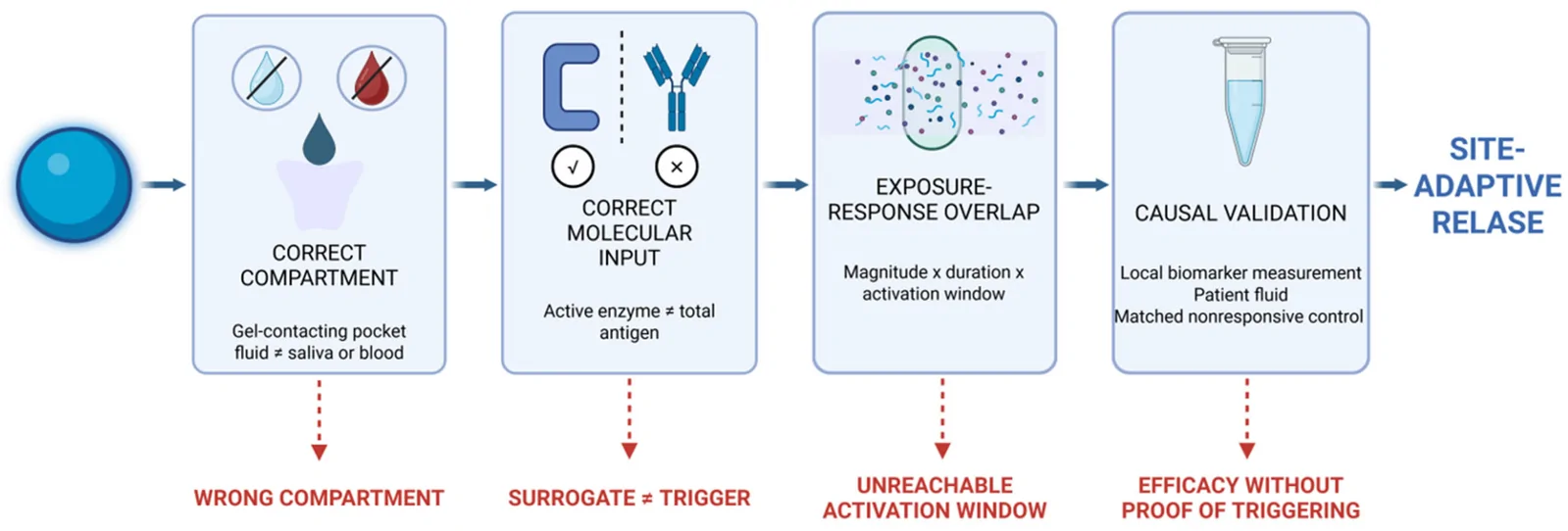
Framework for biological calibration of endogenous stimuli-responsive periodontal gels. Four criteria connect local exposure to cue-dependent material behavior: correct compartment, correct molecular input, exposure–response overlap, and causal validation. Corresponding reporting and validation items are provided in Table 3. Therapeutic efficacy alone does not establish endogenous triggering or clinical precision. Created with BioRender.com.

Biological calibration requires evidence that the complete formulation responds to the molecular input encountered at its intended site of use. Figure 2 summarizes the four criteria, and Table 3 translates them into minimum reporting and validation items. Table S1 applies a simplified version to the systems in Table 2. Figure S1 compares selected human periodontal pH and GCF glucose measurements with material challenge conditions, distinguishing observed ranges and study-specific means from discrete test levels and a reported material test span.

The checklist separates documentation of an experiment from evidence that the formulation operates under biologically relevant conditions. A response at two widely separated challenge values leaves the intervening response curve unresolved. Similarly, nominal concentration alone cannot describe exposure when a cue is continuously generated, buffered, or cleared. Response onset, latency, and persistence therefore need to be interpreted within the intended deployment setting; a broadly explored test range should not be described as physiological when human exposure remains uncertain.

Calibration applies to the finished depot because carrier composition and rheology influence retention, biofilm delivery, and release [116]. In a liposomal chitosan hydrogel developed for MRSA skin infection, liposome incorporation altered viscosity, texture, and azithromycin release [117]. This provides a formulation-level precedent, while wet adhesion and swelling impose additional periodontal constraints [118]. Embedding a responsive nanoparticle or liposome in a gel can therefore change the mechanics and transport governing its response. Component-level responsiveness may not persist in the final formulation, and nominal release does not guarantee exposure at the target surface.

Limited fluid availability makes staged validation necessary: single-site GCF volumes are often submicroliter. Microdroplet or microfluidic assays and standardized dilution with recovery controls can support initial testing, while reconstructed GCF can help identify matrix effects before native-fluid experiments. Pooled samples may support discovery but cannot establish between-site reproducibility. Changing a responsive group may alter network properties and baseline release, making exact comparator matching difficult. Complementary cell-free tests can use verified cleavage-resistant peptides or selective inhibitors for protease-responsive gels; catalase for H_2_O_2_-responsive boronates; and controlled pH and ionic strength with free-drug controls for pH-responsive systems. GOx systems can include inactive-enzyme controls while monitoring pH, peroxide, and oxygen. Release or degradation should be measured directly, with inhibitor- or scavenger-only controls included in biological assays. These tests should complement nonresponsive comparators where feasible, with residual differences in network properties, drug loading, and baseline release reported (Table 3).

Biological calibration and therapeutic sufficiency remain separate questions. A potential “responsiveness paradox” arises if release falls as inflammation resolves: reduced dosing may suit some host-modulating therapies, but antimicrobial delivery could decline before residual biofilm is controlled. The implications depend on payload pharmacodynamics, treatment duration, and release that persists independently of triggering. Testing under declining cue levels should therefore establish whether effective local exposure is maintained for the intended treatment period, alongside biofilm recovery or wound-healing outcomes appropriate to the payload.

Translation can proceed in stages. A thermoreversible doxycycline/lipoxin A4 gel tested in naturally occurring canine periodontal pockets illustrates evaluation of delivery and pharmacological activity before adding an endogenous adaptive-release claim [119]. Early human studies could then test whether measured site biology predicts material behavior and local exposure following standard instrumentation. Retention and local pharmacokinetics would establish whether the depot remains long enough for its response kinetics to matter, with safety and periodontal outcomes providing clinical context. This mechanistic link would support subsequent trials of the added therapeutic value of responsiveness.

## 12. Regulatory and Manufacturing Considerations

The regulatory pathway for responsive periodontal gels depends on their intended therapeutic action and the functions of their constituents. In the United States, formulations incorporating drug and device constituents may qualify as combination products, with the primary mode of action determining the lead FDA reviewing center [120]. In the European Union, a medical device incorporating an integral medicinal substance with ancillary action is classified as class III under Rule 14 of Regulation (EU) 2017/745. When the medicinal substance provides the principal action, the integral product is governed by medicinal-product legislation, while relevant device safety and performance requirements remain applicable [121]. Classification therefore requires assessment of the individual formulation and its intended use.

Biological evaluation of device constituents should follow a risk-based approach informed by ISO 10993-1:2025 [122] and the complementary dental standard ISO 7405:2025 [123]. For periodontal gels, the assessment should reflect the contacted tissues, exposure duration, and degradation profile. It should address the final drug-loaded formulation, including residual crosslinkers, released constituents, and degradation products; cytocompatibility of the unmodified parent polymer alone is insufficient. For gels that form or degrade in situ, evaluation at appropriate stages of polymerization and degradation should characterize the species encountered during treatment [124].

Microbiological specifications should reflect the formulation, placement route, and regulatory category. For devices supplied sterile, the sterilization method and its validation should be documented within the applicable regulatory pathway [125]. For sterile medicinal products, EMA guidance favors terminal sterilization whenever feasible, with justification of alternatives such as aseptic processing [126]. For responsive gels, we recommend assessing whether processing and storage preserve gelation, network integrity, drug activity, and stimulus-dependent release. Calibration against periodontal exposures should therefore include the final formulation after the intended manufacturing process and representative storage conditions.

## 13. Future Perspectives

The next advance is likely to come from narrowing the intended use. Immediate placement after instrumentation, treatment of a residual pocket during maintenance, and filling of a surgically exposed intrabony defect require different residence times, tissue compatibility, and release programs. Development should begin with one setting and the simplest material architecture capable of meeting its exposure requirements. A second or third responsive component should provide a measurable gain in selectivity or therapeutic exposure that justifies additional synthesis, stability, and manufacturing variability.

A shared periodontal exposure atlas could link site-level measurements to treatment timing, collection method, and analytical uncertainty. It should preserve distributions and paired trajectories rather than reduce them to a single disease threshold. Reference materials with known response curves could help laboratories distinguish variation in patient fluids from variation in assay performance. Low-volume interface sensors and microfluidic models may support this work, provided that sensing and sampling do not substantially disturb the measured signal.

Ultimately, calibration might enable selection among a small family of validated depots according to a local proteolytic or physicochemical phenotype. This remains a prospective strategy, not an established clinical application. Early translational studies should report inadequate triggering, excessive off-state release, premature erosion, and premature loss of effective exposure as the cue declines, alongside successful responses. Linking these failure modes to manufacturing reproducibility and degradation-product exposure would help identify which systems merit comparative efficacy trials and when an autonomous response offers a practical advantage over established sustained delivery.

## 14. Limitations of the Review

This comprehensive review has limitations. The search was designed for broad integration of heterogeneous periodontal measurements and materials studies rather than formal risk-of-bias scoring. Embase, Web of Science, and Scopus were not systematically searched, which may have reduced coverage of materials science and engineering studies despite citation chaining and targeted supplementary searches. Human evidence was often based on small, historical, or methodologically diverse studies, and comparisons with material tests were indirect. Complete primary full texts were unavailable for three of the 15 material studies in Table 2 [14,15,16]; assessment was limited to accessible abstracts, publisher text, and verifiable original figures reproduced in subsequent publications, as specified in Table S2. Therefore, the absence of information in the available reports should not be interpreted as evidence that these experiments were not conducted or reported in the original studies. Similarly, the absence of a reliable human range should be interpreted as an evidence gap rather than as evidence that a given cue lacks biological relevance.

The calibration categories are descriptive and depend on the available evidence, which may change as new compartment- and treatment-matched data emerge. The comparisons are hypothesis-generating and should not be interpreted as validated physiological thresholds or a formal ranking of material quality.

## 15. Conclusions

Stimuli-responsive periodontal gels have advanced rapidly, but biological calibration remains uneven. Human pocket pH ranges from mildly acidic to alkaline and does not support a universal acidic or alkaline switch. Proteolysis offers the strongest mechanistic route because active enzymes provide catalytic amplification, yet antigen, active immunoreactivity, and crosslink-cleaving activity are not interchangeable. The cited MMP-8 PEG gel demonstrates response to sustained recombinant enzyme but has not been connected to a native-GCF activation range. ROS-responsive systems face a different gap: most human studies measure damage or antioxidant balance, whereas material tests use defined H_2_O_2_ challenges that do not reproduce buffered pocket exposure directly.

Oxygen, H_2_S, microbial metabolites, and glucose further show why calibration cannot be reduced to one matched value. Oxygen tension has been measured directly, but oxygen-generating gels do not sense pO_2_. For H_2_S, numerical overlap remains provisional because equivalence between historical sulfide measurements and the material-active input is unestablished. Human SCFA measurements show disease and treatment associations, but periodontal trigger transduction remains little explored. Some glucose challenges overlap reported human GCF values, yet exposure in defined diabetic periodontal sites and the coupled effects of GOx on oxygen, pH, and peroxide remain insufficiently characterized.

Most systems reviewed are preclinical gels that respond under selected experimental conditions. Progress toward site-adaptive treatment requires evidence that local cues govern material behavior and produce therapeutically relevant drug exposure, assessed using the framework in Figure 2 and Table 3. Biological calibration should therefore guide material optimization: it determines whether the formulation can perform its claimed adaptive function at the intended periodontal site and during the relevant treatment phase.

Material chemistry and translation should be evaluated together. The polymer backbone, responsive junction, crosslink density, and payload interaction determine how a measured cue becomes a release response, while network disassembly alone does not establish complete biodegradation or clearance. A clinically credible formulation must retain its calibrated behavior after manufacture, sterilization where required, and storage, with biological safety demonstrated for both the finished depot and the products generated during use.

## Figures and Tables

**Table 1:** Candidate endogenous cues: human evidence and design priorities.

Candidate Cue	Material-Relevant Input	Representative Human Evidence	Design Priority	Study Context and Methods
pH	Interfacial pH	Human values span acidic to alkaline; the largest in situ study reported a mean of 6.92 ± 0.03 [42,45,46,47]	Map interface-specific ranges	1996; 16 participants; in situ iridium-oxide electrode [42] 1991; 69 participants; in situ glass microelectrodes [45] 1983; 5 patients, 47 sites; in situ HIS-FET sensor [46] 2025; 15 periodontitis patients/15 controls; sulcular pH strips [47]
MMP-8	Active, network-accessible enzyme	Patient-level aMMP-8 immunoreactivity: 3.2–23.7 ng/mL; assay-dependent [48,49,50]	Test native-GCF cleavage	2012; 9 female patients; GCF filter strips; monoclonal-antibody ELISA [48] 2010; 10 participants, 83 GCF samples; paper strips; IFMA, dipstick, dentoAnalyzer and ELISA [49] 2014; 40 periodontitis patients/20 controls; extra-sulcular GCF collection; ELISA [50]
MMP-9 and elastase	Crosslink-cleaving activity	MMP-9 variable; elastase activity increased in gingivitis from 481 to 1444 µU/µL [51,52,53]	Use as specificity controls	2014; 83 analysed participants; pooled GCF strips; antibody array [51] 2025; 413 participants in tabulated analytical groups; GCF strips; multiplex bead immunoassays [52] 2001; 12 volunteers; GCF paper strips; fluorometric activity assay [53]
Gingipains	Active Rgp or Kgp	Rgp up to 1.5 µM; Kgp detected infrequently [54,55]	Stratify *P. gingivalis*-rich sites	2013; 18 periodontitis patients/5 controls; crevicular washing; ELISA [54] 2011; 36 patients, 144 sites; paired comparisons of strips, points and washing; sandwich ELISA [55]
H_2_O_2_	Free H_2_O_2_ or interfacial flux	Pilot H_2_O_2_ mean 11.19 µM; separate GSH mean 1.18 mM [56,57]	Model antioxidant buffering	2024; 31 participants, including 12 with periodontitis; GCF paper strips; Amplex Red/peroxidase [56] 2002; 10 periodontitis patients/10 controls; pooled GCF paper strips; HPLC [57]
pO_2_	Local oxygen tension	5–27 mmHg; mean 13.3 mmHg [58]	Validate true pO_2_ sensing	1984; 26 patients, 111 untreated pockets; in situ membrane-covered electrochemical oxygen electrode [58]
H_2_S	Dissolved H_2_S	Detected in 61/79 pockets; historical maximum 1.9 mM [59]	Define population distribution	1992; 33 participants, 79 sites; GCF paper strips; acidified headspace gas chromatography with flame-photometric detection [59]
Propionate and butyrate	Dissolved SCFAs	Propionate 10.23 and butyrate 2.63 mM in stage III/IV periodontitis; higher in deeper/severe disease [60,61]	Develop transduction chemistry	1997; 21 participants; subgingival plaque collected by curette; acid extraction and ion chromatography. [60] 2024; 37 periodontitis patients/19 controls; GCF filter strips; HPCE; samples collected 2008–2011 [61]
Glucose	Native GCF glucose	0.01–0.50 mM (healthy) vs. 0.71–15.4 mM (type 2 diabetes); higher at diseased sites [62,63,64]	Establish activation range	2004; 10 healthy adults; capillary GCF collector; glucose oxidase/peroxidase test tape with optical readout [62] 2005; 10 patients with type 2 diabetes; 200-nL GCF collector; enzymatic fluorometry after elution [63] 2002; 8 periodontitis patients/11 controls; GCF paper strips; glucose oxidase/peroxidase colorimetry [64]
Cytokines and endotoxin	Recognition-accessible analyte	GCF IL-1β in periodontitis: 85.8–882.2 ng/mL; median 17.95 ng/mL in another cohort; endotoxin elevated in aggressive periodontitis [65,66,67]	Develop molecular-recognition transduction	1994; 19 periodontitis patients/14 controls; GCF paper strips; IL-1β ELISA [65] 2018; 147 analysed participants; pooled GCF paper strips; multiplex bead immunoassay [66] 2024; 12 aggressive periodontitis patients/12 controls; GCF paper points; LAL assay [67]

Abbreviations: aMMP-8, active matrix metalloproteinase-8; GCF, gingival crevicular fluid; GSH, glutathione; HIS-FET, hydrogen-ion-sensitive field-effect transistor; HPCE, high-performance capillary electrophoresis; IFMA, immunofluorometric assay; Kgp, lysine-specific gingipain; LAL, Limulus amebocyte lysate; MMP, matrix metalloproteinase; pO_2_, partial pressure of oxygen; Rgp, arginine-specific gingipain; SCFA, short-chain fatty acid. Note: Values are study-specific and were not pooled; most measurements preceded periodontal instrumentation. Years denote publication years unless sampling dates are specified. Counts refer to participants unless samples or sites are explicitly indicated.

**Table 3:** Proposed minimum reporting and validation checklist for biological calibration of responsive periodontal gels.

No.	Domain	Item	Minimum Reporting and Validation Items
1	Correct compartment	1.1	Identify the proposed cue, responsive component, and intended output of the complete formulation (e.g., degradation, release, or conversion).
		1.2	Specify the gel-contacting compartment or interface, intended periodontal condition, and deployment setting.
		1.3	Report placement and sampling times relative to instrumentation, irrigation, ligature removal, or other procedures that alter exposure.
2	Correct molecular input	2.1	Identify the measured chemical species, molecular form, or functional activity and its relevance to the responsive chemistry.
		2.2	Report sampling, assay, units, dilution/normalization, and numbers of participants and sites/specimens; distinguish these from biological and technical replicates in material tests.
		2.3	Identify the human exposure source and clinical/treatment context, distinguishing external evidence from measurements within the material study.
3	Exposure–response overlap	3.1	Report challenge values, exposure durations, and delivery/replenishment schedules; distinguish individual from combined cues.
		3.2	Report paired response magnitude, kinetics, and baseline release; identify onset or an activation window only when experimentally established.
		3.3	Describe medium composition, volume, buffering, relevant proteins/inhibitors/antioxidants, and transport conditions (static, agitated, batch replacement, or perfusion).
		3.4	Compare response-producing conditions with study-specific human exposure measurements, including duration where available, and state limits to comparability.
4	Causal validation	4.1	Report complete-formulation testing in patient-derived fluid, with cue measurement and collection, pooling, dilution, and storage details.
		4.2	Describe cue-specific perturbations and competing-input controls, such as selective inhibitors, scavengers, inactive enzymes, or pH clamping.
		4.3	Describe a chemistry-matched nonresponsive comparator where feasible; disclose residual differences in composition, swelling, mechanics, loading, and baseline release.
		4.4	Distinguish local in vivo cue measurement from evidence linking the cue to material behavior; report local payload exposure over time and retained biological activity where measured.

Note: Author-proposed and not externally validated; items are not scored or weighted. Reporting completeness does not establish calibration or therapeutic efficacy. Identify supporting sources or experiments for each item. Distinguish information not reported in an assessed source from information not verifiable from accessible sources; reserve ‘not tested’ for explicitly unperformed experiments and justify ‘not applicable’.

## Data Availability

No new data were created or analyzed in this study. Data sharing is not applicable to this article.

## Supplementary Materials

The following supporting information can be downloaded at: https://www.mdpi.com/article/10.3390/gels12100897/s1, Figure S1: Human exposure versus material challenge conditions; Table S1: PubMed search strategies; Table S2: Biological calibration checklist applied to periodontal gels.

## Abbreviations

The following abbreviations are used in this manuscript:

aMMP-8	Active matrix metalloproteinase-8
CAT	Catalase
CGRP	Calcitonin gene-related peptide
GCF	Gingival crevicular fluid
GOx	Glucose oxidase
GSH	Glutathione
HA	Hyaluronic acid
HIS-FET	Hydrogen-ion-sensitive field-effect transistor
HPCE	High-performance capillary electrophoresis
IFMA	Immunofluorometric assay
LAL	Limulus amebocyte lysate
MMP-8	Matrix metalloproteinase-8
MMP-9	Matrix metalloproteinase-9
PEG	Poly(ethylene glycol)
pO_2_	Partial pressure of oxygen
PTB	N-phenacylthiazolium bromide
PVA	Poly(vinyl alcohol)
RgpA	Arginine-specific gingipain A
ROS	Reactive oxygen species
SCFAs	Short-chain fatty acids
ZIF-8	Zeolitic imidazolate framework-8
