# Supplementary material for: From Biomarker to Trigger: Biological Calibration of Stimuli-Responsive Gels Toward Precision Periodontal Therapy
Source: Gels. 2026 Oct 3;12(10):897. doi: 10.3390/gels12100897 (PMC13704768; doi:10.3390/gels12100897)
Supplement: Supplementary file 1 [file gels-12-00897-s001.zip › Table S1.pdf]

Search strategies

Search coverage: through 22 August 2026, with targeted additions through 20 September 2026.

Filters: The PubMed search was limited to records indexed as English. No restrictions were applied regarding publication date, study design, species, age, or publication type.

Additional sources: The manuscript reports backward and forward citation searching, targeted Google Scholar and publisher searches, and consultation of official regulatory and standards sources. Embase, Web of Science, and Scopus were not systematically searched.

Main combinations. A AND B identifies candidate gel systems. A AND C1 through A AND C9 identify cue-specific evidence independently of gel terminology. A AND B AND C1 through A AND B AND C9 provide focused searches for materials using each cue. The complete search strings were generated by expanding each parenthesized block exactly as shown and combining the blocks according to the combinations listed above.

Table S1. PubMed search blocks and combinations

ID	Concept	Query block
A	Periodontal context	(periodont*[tiab] OR subgingiv*[tiab] OR gingivit*[tiab] OR "gingival crevicular fluid"[tiab] OR "gingival fluid"[tiab] OR "gingival crevice"[tiab] OR "gingival sulcus"[tiab] OR GCF[tiab])
B	Gel and responsive-material terms	(hydrogel*[tiab] OR nanogel*[tiab] OR thermogel*[tiab] OR gel[tiab] OR gels[tiab] OR "stimuli-responsive"[tiab] OR "stimulus-responsive"[tiab] OR "microenvironment-responsive"[tiab])
C1	pH	(pH[tiab] OR acid*[tiab] OR alkalin*[tiab] OR microelectrode*[tiab] OR ISFET[tiab])
C2	Host proteases	("matrix metalloproteinas*"[tiab] OR MMP[tiab] OR "MMP-8"[tiab] OR "MMP-9"[tiab] OR elastas*[tiab] OR collagenas*[tiab] OR gelatinase*[tiab] OR "protease activity"[tiab])
C3	Gingipains	(gingipain*[tiab] OR Rgp[tiab] OR RgpA[tiab] OR RgpB[tiab] OR Kgp[tiab] OR "arginine-specific protease"[tiab] OR "lysine-specific protease"[tiab] OR "trypsin-like"[tiab])
C4	Redox and antioxidant buffering	("reactive oxygen species"[tiab] OR ROS[tiab] OR "hydrogen peroxide"[tiab] OR H2O2[tiab] OR oxidant*[tiab] OR antioxidant*[tiab] OR glutathione[tiab] OR redox[tiab])
C5	Oxygen	(oxygen[tiab] OR hypoxi*[tiab] OR anoxi*[tiab] OR pO2[tiab])
C6	Sulfide	(sulfid*[tiab] OR sulphid*[tiab] OR H2S[tiab] OR "volatile sulfur"[tiab] OR "volatile sulphur"[tiab] OR "methyl mercaptan"[tiab] OR methanethiol[tiab])
C7	Short-chain fatty acids	("short-chain fatty acid*"[tiab] OR "short-chain carboxylic acid*"[tiab] OR "volatile fatty acid*"[tiab] OR SCFA*[tiab] OR propionat*[tiab] OR butyrat*[tiab])
C8	Glucose	(glucose[tiab] OR glycem*[tiab] OR glycaem*[tiab] OR hyperglyc*[tiab] OR diabet*[tiab])
C9	Cytokines and endotoxin	(cytokine*[tiab] OR interleukin*[tiab] OR "tumor necrosis factor"[tiab] OR "tumour necrosis factor"[tiab] OR lipopolysaccharide*[tiab] OR endotoxin*[tiab] OR LPS[tiab])

Note: [tiab] denotes the PubMed Title/Abstract field, including author keywords; * indicates truncation. Each complete query is obtained by substituting the parenthesized blocks into the stated combination. PubMed syntax reference: https://pubmed.ncbi.nlm.nih.gov/help/. Additional contextual searches on carrier architecture, gel chemistry/degradation, and plaque composition were performed as needed to inform specific sections but were not part of the core structured search.
