# Supplementary material for: From Biomarker to Trigger: Biological Calibration of Stimuli-Responsive Gels Toward Precision Periodontal Therapy
Source: Gels. 2026 Oct 3;12(10):897. doi: 10.3390/gels12100897 (PMC13704768; doi:10.3390/gels12100897)
Supplement: Supplementary file 1 [file gels-12-00897-s001.zip › Table S2.pdf]

Table S2. Simplified application of the biological calibration checklist to the responsive periodontal systems in Table 2

System and source access	1. Correct compartment	2. Correct molecular input	3. Exposure-response overlap	4. Causal validation
**PTB-chitosan hydrogel [81]** Main article	Gingival sulcus; placement during ligature induction or, in a separate recovery model, immediately after ligature removal on day 7.	pH; external human pocket-fluid measurements [42,45–47].	pH 5.5, 6.5, and 7.4; release through 288 h. PBS, 37 °C, 120 rpm; scheduled medium replacement. Buffer capacity, medium volume, and controlled flow NR. The response at pH 6.5 overlaps selected human measurements, but differs only modestly from that at pH 7.4.	**Native fluid:** gel activation NR. **Controls:** pH 7.4 and unloaded gel; matched drug-loaded nonresponsive gel NR. **Local cue:** pocket pH NR. ***In vivo* linkage:** pH-dependent release NR.
**Naringin thermogel [79]** Main article	Gel applied to silk before ligature placement in mice; 7 d disease induction, without application after periodontal instrumentation.	pH; external human pocket-fluid measurements [42,45–47]. Formulation pH does not establish pocket pH.	pH 5.5, 6.5, and 7.4; release through 120 h, with late convergence. 37 °C, 120 rpm; scheduled medium replacement. Medium composition, buffer capacity, and controlled flow NR. The early response at pH 6.5 overlaps selected human measurements.	**Native fluid:** gel activation NR. **Controls:** pH 7.4 and unloaded gel; matched drug-loaded nonresponsive gel NR. **Local cue:** pocket pH NR. ***In vivo* linkage:** pH-dependent release NR.
**Embelin dynamic hydrogel [17]** Main article; SI unavailable	Rat periodontal defects; timing relative to periodontal instrumentation NV.	pH; external human pocket measurements [42,45–47]. Lower pH in diseased rat GCF provides animal evidence only.	pH 5.0 versus 7.4; release through 15 d. PBS specified for swelling and degradation. Release medium and replacement protocol NV; controlled flow NR. Human pocket measurements provide limited support for the pH 5.0 release challenge.	**Native fluid:** gel activation NR. **Controls:** free embelin and unloaded gel; matched nonresponsive gel NR. **Local cue:** rat GCF pH measured. ***In vivo* linkage:** pH-dependent release NR.
**ZIF-8/metronidazole hydrogel [82]** Main article; SI unavailable	Periodontal dressing intended for pocket-fluid contact; material and biological testing performed *in vitro*. Application timing not assessed.	pH; external human pocket-fluid measurements [42,45–47].	pH 5.0, 6.5, and 7.5; release through 60 h, including substantial release at pH 7.5. XRD and zinc release support pH responsiveness. Simulated saliva specified for swelling only; release medium and flow conditions NV. The response at pH 6.5 provisionally overlaps human measurements; support for the pH 5.0 challenge is limited.	**Native fluid:** gel activation NR. **Controls:** pH comparisons; matched nonresponsive gel NR. **Local cue:** not assessed. ***In vivo* linkage:** not assessed (*in vitro* study).

System and source access	1. Correct compartment	2. Correct molecular input	3. Exposure-response overlap	4. Causal validation
**Triclosan/deferoxamine polyglycerol nanogel [80]** Main article; SI unavailable	Local treatment in ligature-induced mouse periodontitis; 2 weeks of treatment after 1 week of ligation. Periodontal instrumentation NV.	pH; external human pocket-fluid measurements [42,45–47].	pH 5.5 versus 7.4; release over 24 h. DLS assessed disassembly; HPLC measured released triclosan. Release buffer and replacement protocol NV; controlled flow NR. Human measurements provide limited support for the pH 5.5 challenge in the intended application.	**Native fluid:** gel activation NR. **Controls:** unloaded, single-payload, dual-payload, and TCS-VEA formulations; matched nonresponsive nanogel NR. **Local cue:** pocket pH NR. ***In vivo* linkage:** pH-dependent release NR.
**Rutin–PEG gel [14]** Abstract and publisher text	Cell experiments and local delivery in mouse periodontitis; timing relative to periodontal instrumentation NV.	pH; external alkaline GCF measurements [47,73,74]. Differences in measurement methods and treatment status affect comparability.	pH 6.6-8.2 in phosphate buffer; reduced matrix integrity and increased rutin release reported at higher pH. Response times, buffer capacity, medium replacement, and flow conditions NV. Alkaline GCF measurements provide provisional exposure correspondence.	**Native fluid:** gel activation NV. **Controls:** cue specificity and nonresponsive comparators NV. **Local cue:** pocket pH measurement NV. ***In vivo* linkage:** pH-dependent material response NV.
**MMP-8-PEG gel [19]** Main article; appendix unavailable	Intraoral or periodontal delivery proposed; testing performed *in vitro*. Animal application and exposure after periodontal instrumentation not assessed.	Human MMP-8 immunoreactivity [48–50] does not establish catalytic activity against the gel crosslinks. Recombinant-enzyme concentration therefore does not define an equivalent native-fluid exposure.	PBS without MMP-8, and 10 or 20 nM MMP-8; minocycline/BSA release through 168 h. Figure 4 marks a medium change at 24 h. Complete buffer composition and replenishment protocol NV.	**Native fluid:** gel activation NR. **Controls:** insensitive-peptide degradation comparator, with different swelling; matched drug-loaded release comparator NR. Concentration dependence alone does not establish enzyme specificity. **Local cue:** not assessed. ***In vivo* linkage:** not assessed (*in vitro* study).

System and source access	1. Correct compartment	2. Correct molecular input	3. Exposure-response overlap	4. Causal validation
**RgpA thermogel [15]** Abstract and reproduced original figures	Local delivery in a *P. gingivalis*-associated rat periodontal model; exact placement and timing relative to periodontal instrumentation NV.	Human pocket-wash Rgp measurements [55] do not establish RgpA activity against this network. Substrate accessibility and inhibition in native fluid remain uncertain.	RgpA at 1 and 10 nM; reproduced release curves through 10 d. Enzyme-free PBS/DMEM comparators. SAMP release and early acceleration of SDF-1 release, with substantial baseline SDF-1 release. Complete medium and flow protocols NV.	**Native fluid:** gel activation NV. **Controls:** PEGD@SDF-1 comparator; physicochemical matching as a cleavage-resistant control NV. **Local cue:** pocket RgpA activity NV. ***In vivo* linkage:** cue-dependent activation NV.
**PVA/boronate hydrogel with C5a receptor antagonist and macrophages [22]** Main article and SI	Local periodontal delivery in rats; additional subcutaneous testing. Exposure after periodontal instrumentation in humans not established.	Human GCF and salivary total oxidant status were measured, rather than free H_2O_2. Separate pilot GCF peroxide measurements provide external evidence [56].	100 µM H_2O_2: morphology through 120 h and cell release through 60 h; 1 mM: weight loss through 150 min. Peroxide baths with PBS controls; cell release in culture medium. Native-fluid buffering and controlled flow NR. The available human peroxide measurements do not establish exposure at the gel interface.	**Native fluid:** human GCF oxidant assay; gel activation NR. **Controls:** peroxide-free, stiffness, and payload comparisons; matched nonresponsive gel NR. **Local cue:** rat ROS fluorescence measured. ***In vivo* linkage:** fluorescence and subcutaneous gel disappearance do not establish pocket activation.
**HA/PVA-minocycline/Fe-quercetin hydrogel [18]** Main article and SI	Local delivery in rat periodontitis; post-treatment palatal ROS imaging. Human exposure after periodontal instrumentation not characterized.	Rat DCFH-DA fluorescence is a relative redox measure, rather than free-H_2O_2 quantification. Human bulk-GCF peroxide measurements [56] remain preliminary.	0, 100, and 1000 µM H_2O_2; morphology at 3 h and minocycline release through 4 d. Degradation in PBS; release with 2 mL gel in 5 mL challenge medium. Release buffer, medium replacement, controlled flow, and native antioxidant buffering NR.	**Native fluid:** gel activation NR. **Controls:** peroxide-free, unloaded, and single-/dual-payload gels; matched nonresponsive gel NR. **Local cue:** rat ROS fluorescence measured. ***In vivo* linkage:** paired pocket peroxide and release measurements NR.

System and source access	1. Correct compartment	2. Correct molecular input	3. Exposure-response overlap	4. Causal validation
**CGRP-PVA/boronate hydrogel [97]** Main article; SI unavailable	Diabetic mouse periodontal pockets after ligature removal; weekly injections. Human exposure after periodontal instrumentation not characterized.	H_2O_2; external human evidence comprises preliminary bulk-GCF peroxide measurements [56].	1 mM H_2O_2 versus PBS; degradation through 15 d and CGRP release through 10 d. Complete medium replacement at each sampling point, 37 °C. Renewed batch exposure; controlled flow and native-fluid buffering NR. Comparison with human bulk-GCF measurements is limited by uncertainty in local concentration, flux, and exposure duration.	**Native fluid:** gel activation NR. **Controls:** PBS and unloaded responsive gel; matched nonresponsive gel NR. **Local cue:** free H_2O_2 NR. ***In vivo* linkage:** local CGRP staining supports delivery; causal triggering NR.
**H_2S-responsive OSA/CMCS composite hydrogel [103]** Main article and SI	Local periodontal treatment targeting bacterial H_2S; OSA/CMCS network containing azide-PEG. Timing relative to periodontal instrumentation NR.	Historical sulfide measurements in acidified pocket-fluid samples [59] do not establish exposure to native, gel-accessible H_2S.	Isolated-precursor conversion: 0.01-2 mM H_2S; 1 mM time course through 24 h. Antibacterial tests used precursor pretreated with 2 mM H_2S for 24 h. Whole-gel release: PBS with or without 1 mM H_2S through 14 d, with aliquot replacement. Controlled flow NR. The reported human maximum does not define an exposure distribution.	**Native fluid:** gel activation NR. **Controls:** reacted and unreacted precursors; NOCG retains responsive azide and is not a nonresponsive gel. **Local cue:** pocket H_2S NR. ***In vivo* linkage:** endogenous whole-gel activation in pockets NR.
**Ca-alginate hydrogel-coated GOx/CAT/ZIF-8 system [114]** Main article and SI	Local delivery in diabetic rat periodontitis; crosslinked hydrogel shell around particles. Patient plaque-derived bacteria were cultured before testing, without native pocket-fluid exposure.	Glucose; external GCF measurements of 0.71-15.4 mM in 10 adults with type 2 diabetes [63]. GOx couples glucose exposure to acid and peroxide generation.	5, 15, and 35 mM glucose; release through 90 min. Dialysis: 3 mL sample in 20 mL PBS, pH 7, 37 °C, with sampling and medium replacement. Controlled flow NR. The 5 and 15 mM challenges overlap reported GCF values; 35 mM exceeds that range. Exposure in defined periodontal sites and after instrumentation remains unverified.	**Native fluid:** gel activation NR. **Controls:** glucose-free, enzyme-omission, and proteinase K conditions; enzyme-free CMZ remains acid-responsive. **Local cue:** blood glucose measured; pocket glucose and pH NR. ***In vivo* linkage:** endogenous pocket activation NR.

System and source access	1. Correct compartment	2. Correct molecular input	3. Exposure-response overlap	4. Causal validation
**PBA cationic dynamic hydrogel [115]** Main article and SI	Local injection in diabetic rat periodontitis; gel-retention imaging reported. Human exposure after periodontal instrumentation not characterized.	Glucose, pH, and H_2O_2; external human comparators comprise GCF glucose [63], pocket pH [42,45–47], and pilot GCF peroxide measurements [56].	Separate pH 5.5, glucose 2 g/L (11.1 mM), and 0.1% H_2O_2 challenges versus PBS through 48 h. H_2O_2 percentage basis NV. 1 mL gel in 2 mL challenge medium, 100 rpm; degradation at room temperature. Medium replacement and controlled flow NR. The glucose challenge overlaps reported GCF values in type 2 diabetes, but site-specific exposure remains unverified. Human evidence provides limited support for the pH 5.5 and H_2O_2 challenges.	**Native fluid:** gel activation NR. **Controls:** PBS, unloaded, and single-payload gels; matched non-responsive gel NR. **Local cue:** quantitative pocket inputs NR. ***In vivo* linkage:** retention imaging does not establish cue-dependent activation.
**Glucose/ROS/pH-responsive hydrogel [16]** Abstract and publisher preview	Diabetic periodontal application; exact gel-contacting interface and timing relative to periodontal instrumentation NV.	Reported inputs: glucose, ROS, and pH. External human GCF glucose measurements are available [62–64].	Responses to glucose, ROS, and acidic pH reported, with accelerated degradation and minocycline/GSK2606414 release. Quantitative challenges, response times, release medium, and flow conditions NV. Exposure-response overlap is not assessable without verified challenge values.	**Native fluid:** gel activation NV. **Controls:** cue specificity and nonresponsive comparators NV. **Local cue:** pocket measurements NV. ***In vivo* linkage:** cue-dependent activation NV.

Abbreviations: BSA, bovine serum albumin; CAT, catalase; CGRP, calcitonin gene-related peptide; CMCS, carboxymethyl chitosan; CMZ, calcium-alginate-coated minocycline-loaded ZIF-8; DCFH-DA, 2′,7′-dichlorodihydrofluorescein diacetate; DLS, dynamic light scattering; DMEM, Dulbecco's modified Eagle medium; GCF, gingival crevicular fluid; GOx, glucose oxidase; HA, hyaluronic acid; HPLC, high-performance liquid chromatography; MMP-8, matrix metalloproteinase-8; NR, not reported in the assessed source(s); NV, not verifiable from accessible source(s); OSA, oxidized sodium alginate; PBA, phenylboronic acid; PBS, phosphate-buffered saline; PEG, poly(ethylene glycol); PTB, N-phenacylthiazolium bromide; PVA, poly(vinyl alcohol); RgpA, arginine-specific gingipain A; ROS, reactive oxygen species; SAMP, short antimicrobial peptide; SDF-1, stromal cell-derived factor-1; SI, supporting information. TCS-VEA, vinyl ether acrylate-modified triclosan; TOS, total oxidant status; XRD, X-ray diffraction; ZIF-8, zeolitic imidazolate framework-8. **Note:** The four criteria correspond to Figure 2 and Table 3. This descriptive assessment does not assign scores or rank study quality or therapeutic efficacy. Reference numbers follow the main manuscript.
